# Penile prosthesis in the surgical treatment of Peyronie's disease

**Published:** 2012-09-25

**Authors:** D Ateia, O Voinescu, R Geavlete

**Affiliations:** * MEDAS Medical Center, Bucharest; ** "Prof. Dr. Th. Burghele" Clinical Hospital; *** "Sf. Ioan" Clinical Hospital, Bucharest

**Keywords:** Peyronie’s disease, penile prosthesis, erectile dysfunction

## Abstract

Peyronie's disease appears to be a condition of middle-aged men. In many of them, the disease is accompanied by erectile dysfunction, which, until recently, was attributed to penile deformity. Later, it turned out that the erectile dysfunction has the same causes as in men without Peyronie's disease with vascular and psychological components. In all cases, the pathology of the disease is characterized by the formation of a fibrous plaque, which will lead to penile curvature at different degrees and directions during erection and pain. These symptoms will have a major impact on the sexual life of the patients. The most common, the plaque, is located on the dorsal side of the penis, leading to dorsal deviation. The use of penile prostheses can ensure the development of a normal sex life by correcting the penile deviation and by providing the necessary rigidity for penetration.

The definitive treatment of Peyronie's disease remains controversial. While straightening and grafting procedures have been widely discussed, a part of Peyronie's disease patients do not respond to these surgical procedures because of erectile dysfunction, severe curvature of the penis or a significant reduction in penile length. Procedures for the straightening of the penis in Peyronie's disease are not always successful in restoring penile function. Furthermore, some of the patients, who were surgically treated, developed erectile dysfunction, which required surgical or medical support to perform intercourse. The penile prosthesis implantation in these patients can provide the penile rigidity and straightness required. While discussing the surgical options with the patient, it is important to include the possibility of using a penile prosthesis, which has often had excellent results, low morbidity and the correction of both the penile curvature and the erectile dysfunction. Good results were communicated while using penile prosthesis with or without penile reconstruction. New penile modeling techniques have a high rate of success with a low morbidity rate.

The first attempt of a penile prosthesis was made in 1936 by Bogoras, who used a cartilage fragment as a prosthesis. Later, it switched to semi silicone prostheses (1973), followed by the appearance of inflatable prostheses, that are currently most commonly used. Penile prostheses are divided into three categories: soft, semi-rigid and inflatable.

The most frequent semi-rigid prostheses used are Genesis (Coloplast), AVIS 600 and AMS (**Fig. 1**). This semi-rigid prosthesis does not change in size but the position is memorized. This allows the prosthesis to be inflexible only during sexual intercourse to allow penetration. Semirigid prostheses are cheaper and easier to implant than the inflatable ones because there is no pump or reservoir.

**Fig. 1:**
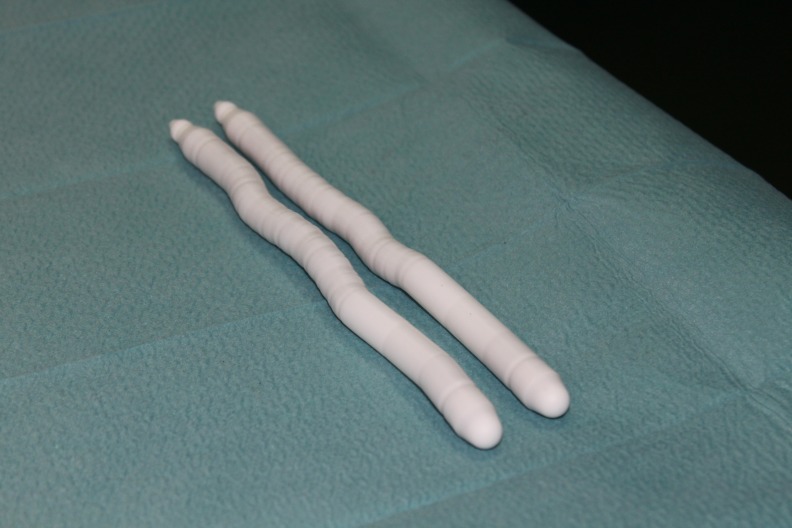
Semirigid prosthesis (AMS Spectra)

Inflatable prostheses are available in models with two or three pieces. In the last years, three-piece inflatable prostheses have been increasingly used, as they improve penile erection and design, functional and cosmetic benefit being certain [**1**]. The models with two pieces are the Excel Resist (Coloplast) and Ambicor (AMS) and are composed of a pair of inflatable cylinders that are placed in the corpus cavernosum and a pump located in the scrotum. When the pump is pressed, the saline solution is introduced into the prosthesis in small reservoirs at the end of each cylinder. This model is used in patients whose health status does not allow the placing of the reservoir in the abdominal cavity.

Prostheses made of three parts consist of two cylinders, scrotal pump and a reservoir that is usually placed under the right abdominal muscle, the Retzius space. Using a thinner cylinder is indicated in patients with severe fibrosis of penile body. To reduce the risk of the prosthesis infection it is wrapped in a layer called InhibiZone [**2**], a combination of rifampicin and minocycline, antibiotics that are released in tissues with a high rate in the initial postoperative period. This layer was especially designed against Staphylococcus Epidermis and Staphylococcus Aureus. 

The selection of the candidates for penile prosthesis takes into account health, presence of penile fibrosis, penile size, previous abdominal surgery and the patient’s desire. In patients with Peyronie's disease, the use of penile prosthesis is justified by a complex deviation that cannot be approached by corporoplasty and grafting, or for increased shortening of the penis. Infections, dermatitis or other lesions in the genital area represent an absolute contraindication to penile prosthesis implant. If patients present balanitis, they will be circumcised and the implantation of the prosthesis will be delayed. Before surgery, prophylactic antibiotics such as a combination of vancomycin and gentamicin are used. The type of surgical approach varies depending on the type of prosthesis used, option of the surgeon, the patient’s anatomy and the past surgical history. In the case of semirigid prostheses, the approach can be subcoronary, infrapubic or penoscrotal. The last two surgical approaches are also recommended when using inflatable prostheses. Penoscrotal incision has the advantage of allowing an easy access to the scrotum, which facilitates a proper positioning of the pump and cylinder. The infrapubic approach allows the reservoir installation. 

**Fig. 2:**
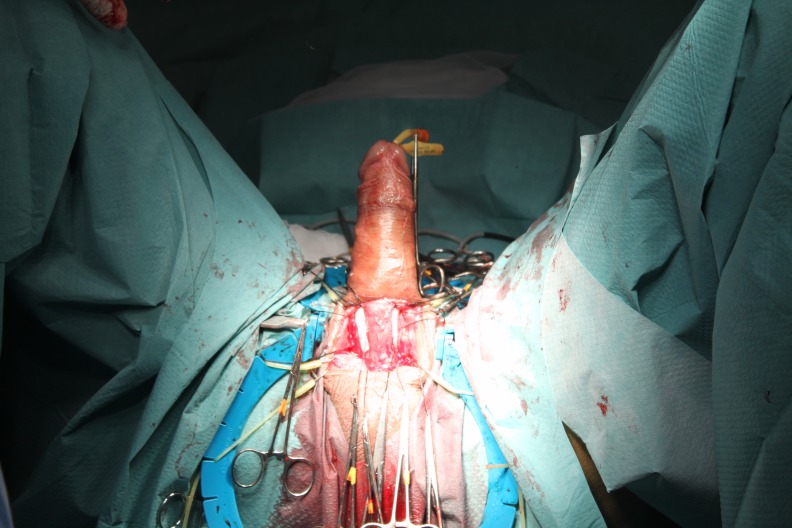
Dilatation of corpus cavernosum

Penile prostheses are implanted in a standard manner by using the penoscrotal approach. A tunnel through the expansion of a Hegar dilator from 7 to 13, in which the prosthesis is placed (**Fig. 2**) is created in each cavernous body. During the expansion, maneuvers of fibrous plaque breaking can be performed. After placing the prosthesis (**Fig. 3**), penile curvature is corrected in most cases (**Fig. 4**). If a curvature remains substantial, a variety of additional techniques can be used: Nesbit corporeal plication, incision or excision with or without graft plate, penile modeling [**3**].

**Fig. 3:**
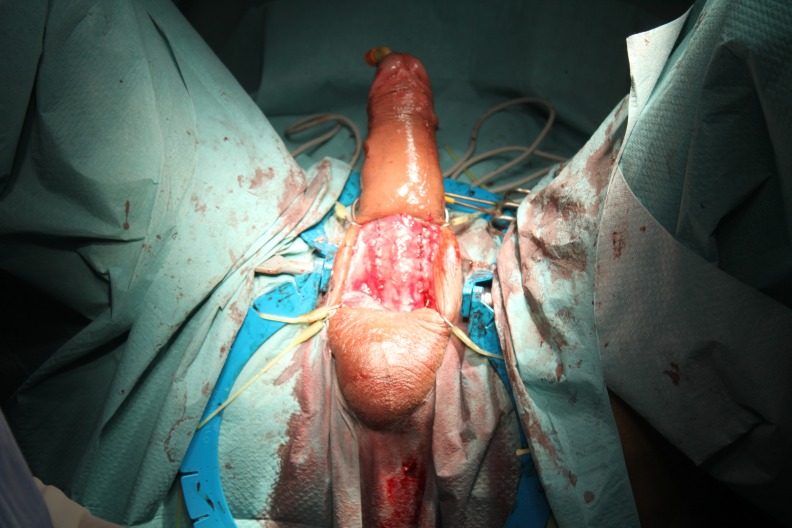
Insertion of prosthesis

**Fig. 4:**
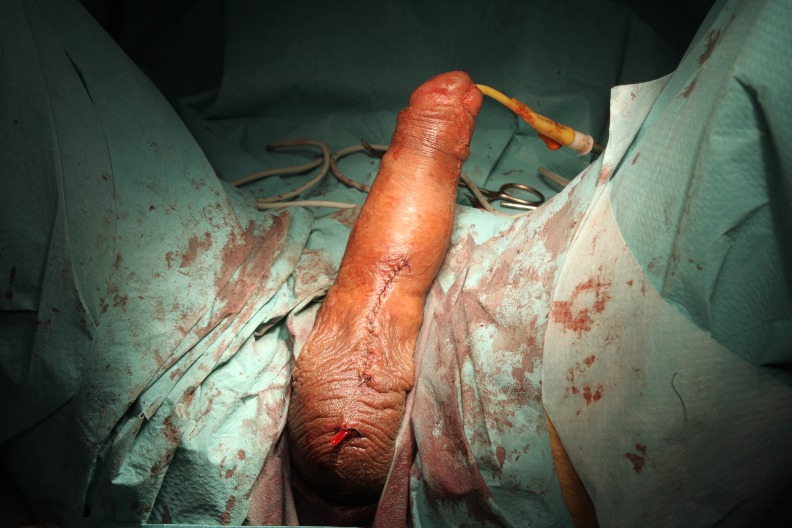
Post operatory result

The most common intraoperatory complication is the perforation of the corpora cavernosum and it occurs in the case of severe fibrosis of the penis. If the urethra is not involved, the procedure can be continued. Post-operative complications are divided into immediate and late complications. The most severe immediate complication is the prosthesis infection, which occurs more often in patients with spinal cord injuries or diabetes [**4**], the incidence ranging from 8-20% [**5**]. In this case, the affected prosthesis is removed and a new prosthesis can be installed immediately or after three months. Using too large cylinders may lead to constant pain and erosion, while the implantation of smaller cylinders can cause the deformation of the penis due to the hypermobility of the glans.

Penile prosthesis, inserted as the first effective treatment for erectile dysfunction since 30 years ago, plays a major role in patients with erectile dysfunction refractory to medical treatment.
